# Effects of *Terminalia catappa* Linn. Extract on *Candida albicans* biofilms developed on denture acrylic resin discs

**DOI:** 10.4317/jced.54776

**Published:** 2018-07-01

**Authors:** Letícia Machado-Gonçalves, Adriano Tavares-Santos, Fábio Santos-Costa, Rafael Soares-Diniz, Livia Câmara-de Carvalho-Galvão, Eduardo Martins-de Sousa, Marco-Aurélio Beninni-Paschoal

**Affiliations:** 1 Post-Graduate Program in Dentistry, CEUMA University, São Luis, Maranhao, Brazil; 2 Post-Graduate Program in Parasite Biology, CEUMA University, Sao Luis, Maranhao, Brazil

## Abstract

**Background:**

Considering the prevalence of denture stomatitis and the challenge of controlling this pathology using conventional therapies, natural products have been suggested as important therapeutic alternatives due to their antifungal and anti-biofilm properties. Thus, this study investigated if immersion in *Terminalia Catappa* Linn. extract (TCE) affects *Candida albicans* biofilms developed on denture acrylic resin discs.

**Material and Methods:**

The minimal inhibitory and minimal fungicidal concentrations (MIC and MFC, respectively) tests were performed for TCE against suspensions of *C. albicans*. For the biofilm assay, discs (10 x 2 mm) were fabricated using a denture acrylic resin with surface roughness standardized. The biofilms were allowed to develop for 24 hours. Then, they were immersed in the following treatments overnight (8 hours): phosphate-buffered saline (PBS, control), TCE at MIC, 5XMIC or 10XMIC. The biofilms were analyzed for cell counts and microscopy. Data were analyzed by ANOVA followed by a Tukey test at a 5% significance level.

**Results:**

The minimal concentration of TCE required to inhibit *C. albicans* was 6.25 mg/mL, while MFC was 12.5 mg/mL. Immersion in TCE at MIC was sufficient to reduce 80% of the biofilm viable cells compared to the control group (*p*< 0.001). Microscopic images confirm that immersion at 5XMIC and 10XMIC had a fungicidal activity with no significant differences between the concentrations regarding viable cells counts (*p*> 0.05).

**Conclusions:**

Within the limitations of this study, it was possible to conclude that immersion in TCE reduced the *C. albicans* biofilms cells developed on the denture acrylic surface.

** Key words:**Terminalia catappa Linn, Biofilm, Candida albicans.

## Introduction

*Candida albicans* is the main microorganism involved in the pathogenesis of denture stomatitis (DS). This pathogen is naturally present in oral cavity flora and is capable of forming a biofilm that can adhere to oral mucosa or acrylic denture surfaces (1,2). The transition from normal commensalism conditions to a parasitism situation may occur when an imbalance between the host and fungus arises, which can contribute to the virulence of *C. albicans* (3). Although this pathology can be asymptomatic, discomfort such as swelling, pain, and burning sensations in the mouth could impair the ingestion of liquids and food, interfering in patients’ quality of life (2-4).

The most widely used hygiene method to control biofilms in dentures is mechanical brushing; although, chemical solutions also could be used as auxiliary methods (5). Antifungal substances such as fluconazole and nystatin are used in cases where DS is recurrent (6). However, the resistance of *C. albicans* to drug compounds is a major

challenge in reducing the propagation of biofilms and could lead to treatment failure. Thus, an antifungal substance with fewer side effects would be of great value in treating oral *C. albicans* (2), considering the problem with controlling DS using conventional therapies (7-9).

In the search for pharmacologically active compounds, natural products have contributed significantly to create

new medicines to use as therapeutic agents (9-11). Among these natural products, *Terminalia catappa* Linn. is emphasized, since it has been investigated in several studies and is characterized as having a diverse chemical composition (12-19). *T. catappa* L. extract exhibits biological activities including antioxidant (12), antiviral (14), anti-inflammatory (15), antimicrobial, and hepato-protective (16). Previous studies suggested their effectiveness against bacteria (17) and fungi (18), but little is known about *T. catappa* L. against *C. albicans*.

Given the lack of scientific information about *T. catappa*

on *C. albicans* and the relevance of discovering new agents for the management of biofilm-dependent diseases, this study aims to investigate the effects of using *T. catappa* L. extract to reduce *C. albicans* biofilms that have formed on denture surfaces. Also, it is important to note that in several studies, the susceptibility tests of *T. catappa* L. was performed in planktonic cells (15-19), while most *C. albicans* cells were associated with biofilms in the oral cavity. Fungi growing in biofilms are more resistant to antimicrobial agents than planktonic cells (6,9).

Therefore, the aim of this study is to evaluate if immersion in TCE has an effect on *C. albicans* biofilms developed on denture acrylic discs. The TCE was investigated

against planktonic cells of *C. albicans* through MIC and MFC and also was evaluated against *C. albicans* biofilms.

## Material and Methods

-Study design

The TCE against *C. albicans* cells was investigated by MIC and MFC. For biofilm analysis, denture acrylic resin discs were fabricated and had their surfaces’ roughness standardized. *C. albicans* biofilms were allowed to develop for 24 hours. The developed biofilms were immersed overnight (8 hours) at PBS (control), TCE at MIC, 5XMIC or 10XMIC. Analyses included cell counting and microscopic assays. Tests were performed in triplicate on three moments (N = 9). Data were analyzed with ANOVA, followed by Tukey’s test at 5% of the significance level.

-Botanical collection, identification, and preparation of extract *T. catappa T. catappa* L. leaves (i.e., almond from the beach) was collected from January 2016 to September 2016. For botanical identification, exsiccate was sent to the Ático Seabra Herbarium of the Federal University of Maranhão. The leaves were dried in a greenhouse with air circulation (37°C for 48 hours) and then were triturate in a cutting mill. Approximately 200 g of material was macerated four times with 70% ethanol at room temperature for 24 hours. The extract obtained was filtered, concentrated using a rotatory evaporator, and then liofilized. The liofilized residue was diluted in dimethyl sulfoxide (DMSO) to a concentration of 100 mg/mL, filtered-sterilized using a membrane of 0.22 μg/mL, and maintained at 4oC for 24 hours until use in the experimental assays.

-*C. albicans* growth conditions

An ATCC reference of *C. albicans* (90028) was cultivated in Sabouraud dextrose agar (SDA; Difco, Detroit, USA) for 24 hours at 37°C. Then, a loop of cells was suspended into a yeast nitrogen base culture medium (YNB; Difco, Detroit, USA), supplemented with 100 mM glucose, and incubated at 37°C. In the exponential growth phase (approximately 20 hours), the cells were washed with phosphate-buffered saline (PBS), and the inoculum was standardized using an optical density of 0.25 at a wavelength of 520 nm at a means of ~107 cells/mL.

-Susceptibility tests

The M27-A3 standards were used to define the MIC and MFC tests (20). Fluconazole (Sigma Aldrich Co., St. Louis, USA), PBS, and the solvent DMSO were used as controls. The MIC was determined as the minimal concentration of TCE that was able to inhibit visible cell growth. The non-visible growth was inoculated on SDA and incubated at 37°C for 48 hours for MFC purposes. The MFC was the minimal concentration of TCE that killed ≥ 99% of the fungal cells.

-Antifungal effects on biofilms

Disc fabrication

Discs of 10-mm diameter and 2-mm thickness were fabricated with a denture acrylic resin (VipiCril Plus; Vipicril, São Paulo, Brazil) using a stainless matrix in accordance to the manufacturer’s directions. After, their surfaces were ground in a horizontal polisher (model APL-4; Arotec, Brazil) using 320-, 400-, and 600-grit aluminum oxide papers. The surface roughness was determined by a rugosimeter (Mitutoyo Corp., Tokyo, Japan), where the mean of three measurements was calculated and standardized at 0.36 ± 0.05 μm. The discs were disinfected using 1.0% sodium hypochlorite under agitation for 3 minutes.

Biofilm development

In a 24-well culture plate, each disc received an aliquot of *C. albicans* standard suspension (~107 cells/mL) and was incubated at 37°C. After 24 hours, the biofilms were washed with PBS to eliminate non-adherent cells and were immersed overnight (8 hours) at 37°C in the following treatments: PBS (control), TCE at MIC, 5XMIC or 10XMIC.

Cell counting

Biofilms that formed on the disc surfaces were disrupted by sonication (7 W, for 30 s), and the resulted solution was serially diluted in PBS and inoculated onto SDA (9). After 24 hours of incubation at 37°C, the cells were counted using a stereomicroscope and were transformed to cells/mL.

Microscopic analysis

The biofilms that formed on the disc surfaces were stained by SYTO-9 and propidium iodide (Live/Dead BacLight viability kit; Invitrogen-Molecular Probes, Eugene, USA) for 20 minutes at 37°C and were protected from light. Five randomly optical fields were evaluated with fluorescence microscopy (Axio Imager Z2; Carl Zeiss, Oberkochen, Germany) using a 63.4x immersion lens.

-Statistical analysis

The data were analyzed by the SAS/LAB software package (SAS Software, version 9.0; SAS Institute Inc., Cary, NC, USA), applying a significance level at 5%. The cell counts data were analyzed by one-way ANOVA followed by Tukey’s HSD test, with the immersion treatment considered as a study factor.

## Results

-Susceptibility tests

The analysis revealed that the amount of TCE required to inhibit *C. albicans* planktonic cell growth ranged from 12.5 to 6.25 mg/mL, while the concentration required for fungicidal effects ranged from 25 to 12.5 mg/mL (i.e., MIC and MFC, respectively). The MIC value for fluconazole used as a control group ranged from 1.0 to 0.5 μg/mL.9 The MFC:MIC ratio showed that the TCE was fungicidal against *C. albicans*, since the extracts are fungicidal when the MFC:MIC ratio is between 1:1 and 2:1. This classification was proposed by Hafidh et al. (21) when establishing the nature of the antifungal effect in regard to the inhibition or killing of tested microorganisms (22).

-Antifungal potential in biofilms

**Figure 1:**
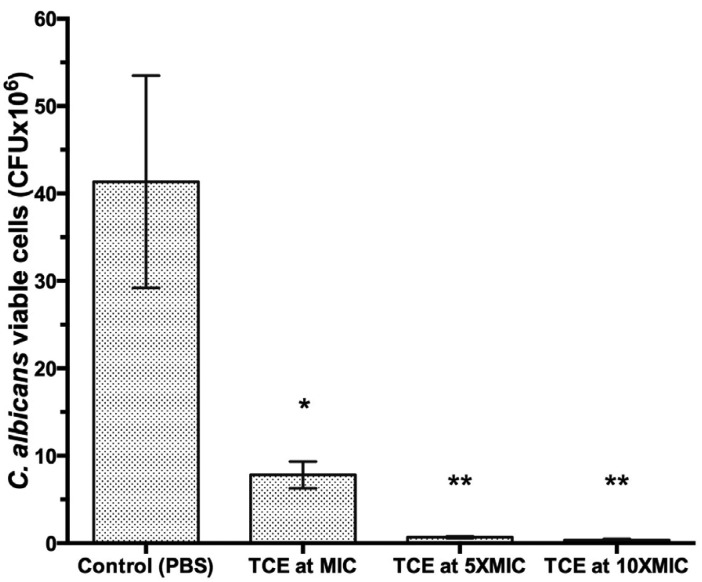
Cell count of C. albicans biofilms developed on denture acrylic discs and were immersed for 8 hours at PBS, TCE at MIC, 5XMIC or 10XMIC. Different symbols (*,**) represent statically significant differences between groups (ANOVA one-way followed by a Tukey test, *p* < 0.05).

Figure 1 shows the development of the *C. albicans* biofilm inhibition after treatment with the TCE extract at different concentrations. Cells/mL measured the growth, and the results showed that exposure of TCE for 8 hours had a significant effect in comparison to the control group (*p* < 0.05), regardless of the concentration used. Immersion at MIC was sufficient to reduce 80% of viable biofilm cells when compared to the control group in PBS (*p* < 0.001).

-Microscopic analysis

**Figure 2:**
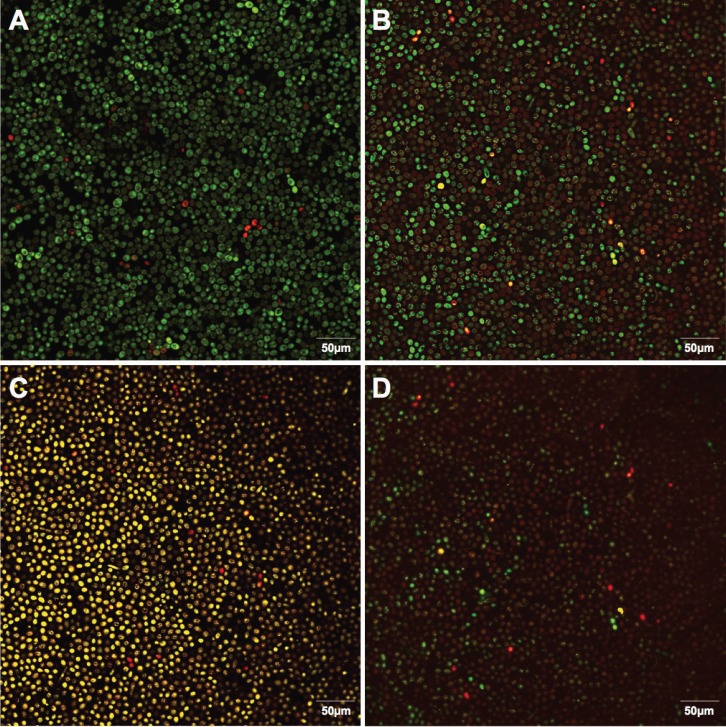
Representative microscope images of C. albicans biofilms developed on denture acrylic discs and immersed for 8 hours at PBS (A), TCE at MIC (B), 5XMIC (C) or 10XMIC (D). SYTO-9 and propidium iodide labeled live and dead cells in green or yellow/red, respectively.

Microscopy revealed that the TCE extracts altered the viability of yeast cells compared to the PBS (Fig. 2), markedly at 10X MIC concentration, which substantially decreased the metabolic activity of the fungal cells. Moreover, microscope images confirmed that immersion

at 5XMIC and 10XMIC have a fungicidal activity, with no significant differences among these concentrations regarding viable cells (*p* > 0.05).

## Discussion

The biological activity of natural extracts against fungal

species has been confirmed by many studies, but the focus is often on antifungal activity against planktonic cells. However, the growing of fungal biofilms present specific characteristics and are more resistant to antimicrobial agents than planktonic cells (6,9,22). Therefore, this study searched for natural products that have anti- biofilm properties and antifungal activity that attack oral microorganisms.

Research exploring the relation between plant products and antifungal properties has been a trend in all fields of fungal control (9-11). *T. catappa* L. is a plant species

that contains a wide variety of components (12-18). However, there are few studies regarding their antifungal properties, and little is known about the anti-Candida activities of extracts obtained from *T. catappa* L. leaves (TCE). The present study revealed important evidence about the antifungal potential of TCE when used as an immersion solution to reduce *C. albicans* biofilms developed on denture acrylic resin surfaces.

The MIC and MFC tests showed that the TCE has a fungistatic action at 6,25 mg/mL against planktonic cells and a fungicidal activity at 12,5 mg/mL. A classification for the antimicrobial activity of natural products (23) has been established: strong inhibitors, MIC up to 0.5 mg/mL; moderate inhibitors, MIC between 0.6 and 1.5 mg/mL; and weak inhibitors, MIC above 1.6 mg/mL. Considering this, the TCE was ranked as a weak inhibitor, regardless of the good results. The effects of the TCE against planktonic cells had been established earlier (18), and the presented data are in accordance with the literature. However, considering that Candida cells are associated with biofilms in the oral ambience and that biofilms have a higher antifungal resistance (6-8), investigations about the behavior of biofilms when exposed to TCE are of great importance.

The immersion of a biofilm that formed over an acrylic denture surface in a TCE solution was conducted o mimic an overnight denture-soaking period of 8 hours (9). These results revealed that immersion in TCE at MIC was sufficient to reduce viable cells. Recently, hydrolyzable tannins, glycosylated phenols (especially C-flavonoid glycosides) and gallic acid were found after a chemical analysis of *T. catappa* L. (18). Hydrolyzable tannins are associated with the antioxidant activities related to this plant (16,24) and possibly with antimicrobial activities toward bacteria and fungi (25-27). In addition, glycosylated phenols and gallic acid also may contribute to its antifungal activity (18). Previous research shows that gallic acid obtained from other plants has an antimicrobial potential (28). Glycosylated phenolic, specifically phenolic acids and C-flavonoid glycosides, have shown promising activity against Candida species (29-30). The increased number of dead cells stained in yellow/red observed in the microscopic images may confirm the potential fungicidal effects of TCE, probably due to its bioactive compounds. Future investigations should analyze mechanisms that explain the fungal cell death.

Additionally, the good results observed when TCE is used as an immersion solution against *C. albicans* biofilms, it is important to note that investigation about the safety of TCE when in contact with human mucosa and the possible effects on physical and mechanical properties of acrylic surfaces (9), is of utmost importance when analyzing the possibility of the herb as an auxiliary treatment for CADS. Nevertheless, further research should explore the possible toxic effects of TCE against human cells and the effects on the mechanical or physical properties of denture acrylic resin. Based on these and other results, TCE emerges as a promising candidate for nonclinical and clinical toxicology testing for the development of new drug treatments of DS.

## Conclusions

Within the limitations of this study, it was possible to conclude that immersion in TCE reduced *C. albicans* biofilms cells developed on denture acrylic surface. These results demonstrate the potential of using *T. catappa* L. as an alternative substance to control *C. albicans* biofilms on denture acrylic surfaces.
